# Association of Maternal Perinatal SARS-CoV-2 Infection With Neonatal Outcomes During the COVID-19 Pandemic in Massachusetts

**DOI:** 10.1001/jamanetworkopen.2021.7523

**Published:** 2021-04-23

**Authors:** Asimenia Angelidou, Katherine Sullivan, Patrice R. Melvin, Jessica E. Shui, Ilona Telefus Goldfarb, Ruby Bartolome, Neha Chaudhary, Ruben Vaidya, Ivana Culic, Rachana Singh, Diana Yanni, Silvia Patrizi, Mark L. Hudak, Margaret G. Parker, Mandy B. Belfort

**Affiliations:** 1 Department of Neonatology, Beth Israel Deaconess Medical Center, Harvard Medical School, Boston, Massachusetts; 2 Precision Vaccines Program, Division of Infectious Diseases, Boston Children’s Hospital, Boston, Massachusetts; 3 UMass Memorial Health Center, University of Massachusetts Medical School, Worcester; 4 Center for Applied Pediatric Quality Analytics, Boston Children’s Hospital, Harvard Medical School, Boston, Massachusetts; 5 Department of Pediatrics, Division of Neonatology and Newborn Medicine, Massachusetts General Hospital, Harvard Medical School, Boston; 6 Division of Maternal Fetal Medicine, Department of Obstetrics and Gynecology, Massachusetts General Hospital, Harvard Medical School, Boston; 7 Department of Pediatrics, Boston Medical Center, Boston University School of Medicine, Boston, Massachusetts; 8 Department of Pediatrics, University of Massachusetts Medical School-Baystate, Springfield; 9 Division of Newborn Medicine, Tufts Children’s Hospital, Tufts University School of Medicine, Boston, Massachusetts; 10 Department of Pediatric Newborn Medicine, Brigham and Women’s Hospital, Harvard Medical School, Boston, Massachusetts; 11 Department of Pediatrics, University of Florida College of Medicine, Jacksonville

## Abstract

**Importance:**

The incidence of mother-to-newborn SARS-CoV-2 transmission appears low and may be associated with biological and social factors. However, data are limited on the factors associated with neonatal clinical or viral testing outcomes.

**Objective:**

To ascertain the percentage of neonates who were born to mothers with positive SARS-CoV-2 test results during the birth hospitalization, the clinical and sociodemographic factors associated with neonatal test result positivity, and the clinical and virological outcomes for newborns during hospitalization and 30 days after discharge.

**Design, Setting, and Participants:**

This multicenter cohort study included 11 academic or community hospitals in Massachusetts and mother-neonate dyads whose delivery and discharge occurred between March 1, 2020, and July 31, 2020. Eligible dyads were identified at each participating hospital through local COVID-19 surveillance and infection control systems. Neonates were born to mothers with positive SARS-CoV-2 test results within 14 days before to 72 hours after delivery, and neonates were followed up for 30 days after birth hospital discharge.

**Exposures:**

Hypothesized maternal risk factors in neonatal test result positivity included maternal COVID-19 symptoms, vaginal delivery, rooming-in practice, Black race or Hispanic ethnicity, and zip code–derived social vulnerability index. Delivery indicated by worsening maternal COVID-19 symptoms was hypothesized to increase the risk of adverse neonatal health outcomes.

**Main Outcomes and Measures:**

Primary outcomes for neonates were (1) positive SARS-CoV-2 test results, (2) indicators of adverse health, and (3) clinical signs and viral testing. Test result positivity was defined as at least 1 positive result on a specimen obtained by nasopharyngeal swab using a polymerase chain reaction–based method. Clinical and testing data were obtained from electronic medical records of nonroutine health care visits within 30 days after hospital discharge.

**Results:**

The cohort included 255 neonates (mean [SD] gestational age at birth, 37.9 [2.6] weeks; 62 [24.3%] with low birth weight or preterm delivery) with 250 mothers (mean [SD] age, 30.4 [6.3] years; 121 [48.4%] were of Hispanic ethnicity). Of the 255 neonates who were born to mothers with SARS-CoV-2 infection, 225 (88.2%) were tested for SARS-CoV-2 and 5 (2.2%) had positive results during the birth hospitalization. High maternal social vulnerability was associated with higher likelihood of neonatal test result positivity (adjusted odds ratio, 4.95; 95% CI, 1.53-16.01; *P* = .008), adjusted for maternal COVID-19 symptoms, delivery mode, and rooming-in practice. Adverse outcomes during hospitalization were associated with preterm delivery indicated by worsening maternal COVID-19 symptoms. Of the 151 newborns with follow-up data, 28 had nonroutine clinical visits, 7 underwent SARS-CoV-2 testing, and 1 had a positive result.

**Conclusions and Relevance:**

The findings emphasize the importance of both biological and social factors in perinatal SARS-CoV-2 infection outcomes. Newborns exposed to SARS-CoV-2 were at risk for both direct and indirect adverse health outcomes, supporting efforts of ongoing surveillance of the virus and long-term follow-up.

## Key Points

**Question:**

What are the test result positivity rate and health outcomes of maternal SARS-CoV-2 infection among perinatally exposed newborns?

**Findings:**

In this cohort study of 255 neonates born to women with positive SARS-CoV-2 test results within 2 weeks before and 72 hours after delivery, 88.2% of newborns were tested for the virus during the birth hospitalization and 2.2% had positive results. A main risk factor for neonatal test result positivity was maternal social vulnerability, and the burden of SARS-CoV-2 exposure on newborn health was associated with preterm delivery, which was prompted by worsening maternal COVID-19 illness.

**Meaning:**

Results of this study indicate that neonates who were perinatally exposed to SARS-CoV-2 can sustain adverse health outcomes both directly (as evidenced by higher test result positivity rates when born to socially vulnerable mothers) and indirectly (given the sequelae of preterm birth).

## Teaser

This cohort study examines the risks, incidence, and outcomes of SARS-CoV-2 among neonates who were exposed to the virus within 2 weeks before and after birth.

## Introduction

Biologically plausible routes of perinatal SARS-CoV-2 transmission include transplacental, contact with infected secretions during delivery and with respiratory droplets after delivery, and breast milk. Low rates of virus positivity in relevant biological specimens^1,2,3^ suggest that perinatal transmission is uncommon, but accumulating evidence indicates that some neonates who are born to mothers with SARS-CoV-2 do obtain positive test results for the virus.^1^ Systematic reviews of case series of mothers with SARS-CoV-2 reported a neonatal test result positivity rate of 3.1% to 9.1%,^4,5,6^ but such reviews included mainly small studies from single centers, limiting the generalizability of the findings and raising the possibility of selection and/or publication bias.^7^ Larger multihospital cohorts from New York City included up to 149 mothers with SARS-CoV-2 and reported a test result positivity rate of less than 1% in neonates.^8,9^ Overall, considerable uncertainty remains about the true incidence of neonatal test result positivity, which can serve as a proxy for perinatal transmission.

Given the low incidence of neonatal test result positivity for SARS-CoV-2, identifying the risk factors associated with its occurrence is challenging. Mothers who present with COVID-19 symptoms may have higher SARS-CoV-2 viral loads and be more likely to transmit the virus than mothers who are identified by routine screening.^10^ Clinical practices that were initially recommended to reduce perinatal transmission included elective cesarean delivery, mother-newborn separation, and temporary suspension of breastfeeding.^11^ Subsequently, based on accumulating evidence, the American Academy of Pediatrics promoted rooming-in and breastfeeding with precautions.^12^ In addition to clinical factors, social adversity may play a role given that the COVID-19 pandemic has disproportionately affected racial/ethnic minority populations. The specific pathways by which social disadvantage might affect mother-to-child transmission of SARS-CoV-2 include differential access to care and clinician bias.^13^ Discrimination also may be a factor in chronic stress, which diminishes antiviral immune responses.^14,15^ To our knowledge, no epidemiologic study to date has examined the risk factors for neonatal test result positivity.

Several studies have reported short-term health outcomes for neonates who were born to mothers with SARS-CoV-2, including the need for neonatal intensive care unit admission and respiratory support,^16,17^ but little is known about the specific factors associated with neonatal clinical or viral testing outcomes beyond the birth hospital discharge. One New York City hospital routinely followed perinatally exposed newborns and found that none had clinically significant signs of SARS-CoV-2 infection and 6 had negative test results.^18^ Also in New York City, a 3-hospital cohort study of 120 neonates who were exposed to SARS-CoV-2 reported 0 positive result from nasopharyngeal swabs taken at 5 to 7 days and 14 days of life during follow-up, and no clinical concerns in telehealth evaluations at 1 month of age were reported.^9^ Although these previous studies did not suggest that substantial clinical illness in newborns commonly followed perinatal exposure to SARS-CoV-2, more data are needed.

Large, geographically defined cohort studies that track newborns after hospital discharge are needed to accurately define the incidence of neonatal test result positivity for SARS-CoV-2 and to identify the factors associated with increased positive test results. Within a statewide cohort, we conducted a cohort study with the following objectives: (1) to ascertain the percentage of neonates who were born to mothers with positive SARS-CoV-2 test results during the birth hospitalization, (2) to identify clinical and sociodemographic factors associated with neonatal test result positivity, and (3) to describe the clinical and virological outcomes for perinatally exposed newborns during the birth hospitalization and 30 days after discharge. We hypothesized that maternal COVID-19 symptoms, vaginal delivery mode, rooming-in practice, racial/ethnic minority status, and social vulnerability would be associated with increased likelihood of neonatal test result positivity for SARS-CoV-2. We also hypothesized that delivery indicated by worsening maternal COVID-19 symptoms would increase the risk for adverse neonatal health outcomes.

## Methods

This cohort study was approved by the institutional review board of each of the 11 participating hospitals, which deemed this study as having minimal risk and could not be performed without the waiver of informed consent. We followed the Strengthening the Reporting of Observational Studies in Epidemiology (STROBE) reporting guideline.

### Study Design, Sites, Population, and Data Sources

This study included 11 academic or community hospitals in Massachusetts (Baystate Medical Center, Baystate; Beth Israel Deaconess Medical Center, Boston; Beverly Hospital, Beverly; Brigham and Women's Hospital, Boston; Boston Medical Center, Boston; Cambridge Health Alliance, Cambridge; Massachusetts General Hospital, Boston; Newton-Wellesley Hospital, Newton; Tufts Children's Hospital, Boston; UMass Memorial Health Center, Worcester; and Winchester Hospital, Winchester). These hospitals are geographically dispersed within the state; serve a racially/ethnically, culturally, and socially diverse population; and capture data on approximately 37 000 births per year or 52% of annual births within the state (eFigure in the Supplement).^19^ We identified all neonates who were born to mothers with a positive result on the nasopharyngeal polymerase chain reaction test for SARS-CoV-2 from 14 days before to 72 hours after delivery, an inclusion criterion of the National Registry for Surveillance and Epidemiology of Perinatal COVID-19 Infection of the American Academy of Pediatrics.^20^ For this analysis, we selected mother-newborn dyads whose delivery and discharge occurred between March 1, 2020, and July 31, 2020.

Eligible dyads were identified at each hospital through local COVID-19 surveillance and infection control systems. All data were collected manually by hospital-based teams from electronic medical records (EMRs) using a standardized case report form, which was developed by the National Registry for Surveillance and Epidemiology of Perinatal COVID-19 Infection.^20^ We used REDCap as the electronic data capture system.^21,22^

### Exposures and Covariates

Hospital data included maternal demographic characteristics; maternal SARS-CoV-2 infection, testing, and treatment; pregnancy and delivery characteristics; and hospital outcomes for mother and newborn. For this study, the participating hospitals also collected data on maternal occupation, zip code of primary residence, and neonatal encounters up to 30 days after the birth hospitalization, including the type of and reason for the encounter as well as any SARS-CoV-2 testing. During the study period, pregnant women were tested for SARS-CoV-2 according to the guidelines of the individual hospitals, most of which had implemented universal screening by April 27, 2020.^23^

We used the primary residence zip code to calculate the Social Vulnerability Index (SVI),^24^ a measure developed by the Centers for Disease Control and Prevention that uses 15 US Census variables to identify socially vulnerable populations. The SVI has 4 themes based on the American Community Survey census estimates for 2015 to 2018: (1) socioeconomic status, including below poverty, unemployment, median income, and no high school diploma; (2) household composition and disability, including age 65 years or older, age 17 years or younger, disability status, and single-parent household; (3) racial/ethnic minority and language status, including speaks English less than well; and (4) housing and transportation type, including multiunit structure, mobile home, crowding, group quarter, and no vehicle. For each census tract, percentile ranks are generated for the 15 individual variables, the 4 themes, and the overall tract ranking. The SVI scores range from 0, representing the lowest, to 1, representing the highest level of vulnerability. We used overall tract ranking as the indicator for geography-based social vulnerability, with 90th percentile or higher being the most vulnerable.

### Outcomes

Primary neonatal outcomes were (1) positive SARS-CoV-2 test result during the birth hospitalization; (2) indicators of adverse health during the birth hospitalization, including preterm birth (<37 weeks) or low birth weight (<2500 g), very preterm birth (<32 weeks) or very low birth weight (<1500 g), delivery room resuscitation (positive pressure ventilation, intubation, and/or chest compressions), continuous positive airway pressure (CPAP) or mechanical ventilation, therapeutic hypothermia, and length of stay; and (3) clinical signs and viral testing, which were identified through EMR documentation of nonroutine health care visits through 30 days after hospital discharge. Test result positivity for SARS-CoV-2 was defined as at least 1 positive result on a specimen obtained by nasopharyngeal swab using a polymerase chain reaction–based method.

### Statistical Analysis

We performed a comparison between neonates with any positive SARS-CoV-2 test results and those with negative test results on the basis of sociodemographic characteristics, pregnancy and delivery factors, and newborn care practices while accounting for hospital clustering using Cochran-Mantel-Haenszel χ^2^ tests for categorical variables. A 2-tailed *P* < .05 was considered statistically significant. For multivariable analyses, we selected covariates for parsimonious models according to a priori hypotheses and associations observed in unadjusted analyses, and we used mixed-effects logistic regression models to account for hospital clustering. Because of the small number of neonates with positive SARS-CoV-2 test results, we used a robust sandwich estimator to estimate odds ratios (ORs) with 95% CIs.

We hypothesized that the following variables would increase the risk of the neonatal test result positivity: maternal symptomatic SARS-CoV-2 infection, vaginal delivery mode, any rooming-in practice, Black race or Hispanic ethnicity, language status (non-English vs English), and SVI of 90th percentile or higher. Furthermore, we hypothesized that delivery prompted by worsening maternal SARS-CoV-2 infection–related clinical status would be associated with indicators of adverse neonatal health outcomes.

If outcome data were missing, the participant was removed from the analysis. We used SAS, version 9.4 (SAS Institute Inc) for all analyses.

## Results

### Birth Hospital Data

From the 11 participating hospitals, we identified 255 neonates who were born to 250 mothers with positive SARS-CoV-2 test results. Maternal demographic characteristics are outlined in Table 1. The mean (SD) maternal age was 30.4 (6.3) years, and 121 (48.4%) were Hispanic mothers of any race, 50 (20.0%) were non-Hispanic White mothers, and 46 (18.4%) were non-Hispanic Black mothers. For the zip code–derived SVI, 68 mothers (27.2%) had an overall score of 90th percentile or higher. A total of 170 mothers (68.0%) were asymptomatic when tested for SARS-CoV-2, which was a consequence of the implementation of universal surveillance testing. Of the 79 women with symptomatic COVID-19, 23 (29.1%) required hospitalization and/or received medication for COVID-19 treatment before delivery. Worsening COVID-19 illness prompted delivery in 23 mothers (9.2%), of which 20 (87.0%) were cesarean deliveries. In 52 of 113 cesarean deliveries (46.0%), rupture of membranes occurred at birth, which limited the exposure of the fetus to maternal genital tract flora before delivery.

**Table 1.** Maternal Demographic and Pregnancy and Delivery Characteristics

Variable	No. (%)
**Maternal demographic characteristics**	
Total No. of women	250
Age, mean (SD), y	30.4 (6.3)
Maternal race/ethnicity	
Hispanic, any race	121 (48.4)
Non-Hispanic	
White	50 (20.0)
Black	46 (18.4)
Asian	14 (5.6)
Other^c^	18 (7.2)
Missing	1 (0.4)
Maternal language	
English	133 (53.2)
Spanish	83 (33.2)
Other	34 (13.6)
Maternal occupation	
Homemaker	107 (42.8)
Health care	29 (11.6)
Service professions	43 (17.2)
Other or unknown^a^	71 (28.4)
SVI, based on zip code	
Percentile ranking (higher = more vulnerable), mean (SD)	
Socioeconomic status theme	65.7 (23.4)
Household composition and disability theme	53.5 (29.6)
Racial/ethnic minority and language status theme	71.4 (25.7)
Housing and transportation type theme	71.4 (22.2)
Overall percentile ranking	70.1 (24.0)
90th Percentile group: most vulnerable	
Overall	68 (27.2)
No. of themes with ≥90th percentile	
None	73 (29.7)
1	72 (29.3)
≥2	101 (41.1)
Days from first positive SARS-CoV-2 test result to delivery, mean (SD)	4.0 (5.2)
Severity of maternal COVID-19 illness	
Asymptomatic	170 (68.0)
Symptomatic	79 (31.6)
Hospitalized and/or received medication for COVID-19 before delivery	23 (29.1)^b^
Sick at home before admission only	56 (70.9)^b^
Unknown (outborn)	1 (0.4)^b^
**Pregnancy and delivery characteristics**	
Total No. of pregnancies	255
Plurality	
Singleton	245 (96.1)
Multiple	10 (3.9)
Route of delivery	
Vaginal	142 (55.7)
Cesarean	113 (44.3)
ROM at delivery	52 (46.0)^b^
Delivery indicated for worsening maternal COVID-19 illness	
Yes	23 (9.0)
Vaginal	3 (13.0)^b^
Cesarean	20 (87.0)^b^
No	232 (91.0)
Vaginal	139 (59.9)^b^
Cesarean	93 (40.1)^b^

Abbreviations: ROM, rupture of membranes; SVI, Social Vulnerability Index.

^a^ Other occupation categories include finance and marketing, manufacturing and engineering, and student.

^b^ The denominator for this variable is a fraction of the total cohort.

^c^ Other includes American Indian or Alaska Native, Native Hawaiian or Pacific Islander.

Neonatal characteristics are outlined in Table 2. The mean (SD) gestational age at birth was 37.9 (2.6) weeks; 13 neonates (5.1%) were small for gestational age (<10th percentile),^25^ and 62 (24.3%) were delivered either at low birth weight or preterm. Among the newborns, 49 (19.2%) required resuscitation at birth, 88 (34.5%) were separated from their mothers, and 152 (59.6%) were directly breastfed. Four neonates (1.6%) had neonatal encephalopathy and underwent therapeutic hypothermia; all 4 had negative SARS-CoV-2 test results, and their mothers had only mild COVID-19 symptoms. We observed 1 newborn death that was secondary to severe hypoxic ischemic encephalopathy; this newborn’s mother had mild SARS-CoV-2 infection–related symptoms, and the newborn had 2 negative SARS-CoV-2 test results at 24 and 48 hours.

**Table 2.** Neonatal Characteristics and Birth Hospitalization Data

Variable	No. (%)
Neonatal characteristics	
Total No. of neonates	255
Gestational age, mean (SD), wk	37.9 (2.6)
Birth weight, mean (SD), g	3116.3 (655.6)
Apgar at 5 min, mean (SD)	8.62 (1.15)
Female sex	131 (51.4)
SGA (<10th percentile)^a^	13 (5.1)
Very preterm birth (<32 wk) or VLBW (<1500 g)	6 (2.4)
Preterm birth (<37 wk) or LBW (<2500 g)	62 (24.3)
Resuscitation at birth	
Drying and stimulation only	202 (79.2)
Oxygen	4 (1.6)
Positive pressure, intubation, and/or chest compressions	49 (19.2)
Neonatal hospital course	
Month of birth	
March	17 (6.7)
April	79 (31.0)
May	88 (34.5)
June	55 (21.6)
July	16 (6.3)
LOS, mean (SD), d	6.2 (11.2)
Disposition	
Home	247 (96.9)
Transfer	7 (2.7)
Died	1 (0.4)
Neonatal signs	
None	215 (84.3)
Respiratory distress	33 (12.9)
Fever (>37.8 °C)	1 (0.4)
Vomiting or diarrhea	1 (0.4)
Hypotonia	5 (2.0)
Maximal respiratory support	
None	205 (80.4)
Oxygen	10 (3.9)
CPAP or mechanical ventilation	40 (15.7)
Diagnoses	
None	187 (73.3)
Routine newborn^b^	16 (6.3)
Congenital anomalies^c^	12 (4.7)
Infection concerns	12 (4.7)
Surfactant deficiency	10 (3.9)
Respiratory issues other than surfactant deficiency^d^	10 (4.0)
Hypoglycemia	14 (5.5)
Encephalopathy	4 (1.6)
Other^e^	4 (1.6)
Intensive care interventions	
None	194 (76.1)
Antibiotics or antivirals (acyclovir)	32 (12.6)
IV fluids	39 (15.3)
Surfactant	11 (4.3)
iNO, pressors or hydrocortisone	4 (1.6)
Therapeutic hypothermia	4 (1.6)
Any SARS-CoV-2 test during the birth hospitalization	
No	30 (11.8)
Yes	225 (88.2)
Tested once	124 (55.1)^f^
Tested more than once	101 (44.8)^f^
Positive result	5 (2.2)^f^
SARS-CoV-2 positive result overall	5 (2.0)
Hospital care practices	
Any rooming-in with mother	167 (65.5)
Direct nursing by mother	138 (82.6)^f^
No rooming-in	88 (34.5)
Direct nursing by mother	14 (15.9)^f^
Any NICU stay	72 (81.8)^f^
Any maternal milk feeding	
Direct nursing	152 (59.6)
Expressed milk fed by mother or another caregiver	78 (30.6)

Abbreviations: CPAP, continuous positive airway pressure; iNO, inhaled nitric oxide; IV, intravenous; LBW, low birth weight; LOS, length of stay; NICU, neonatal intensive care unit; SGA, small for gestational age; VLBW, very low birth weight.

^a^ Calculated per Olsen growth charts.^25^

^b^ Routine newborn diagnoses include prematurity, apnea of prematurity, feeding immaturity, immature thermoregulation, ankyloglossia, hyperbilirubinemia, ABO incompatibility, failed car seat test, hemangioma, cephalohematoma, low resting heart rate, and slow feeding.

^c^ Congenital anomalies include ear pit, tracheoesophageal fistula, hydronephrosis or pyelectasis, and Smith-Lemli-Opitz syndrome.

^d^ Respiratory issues other than surfactant deficiency include delayed transition, pulmonary hypertension, hypoxemia, and desaturation.

^e^ Other includes hypotension, hypothermia, intraventricular hemorrhage, and microcephaly.

^f^ The denominator for this variable is a fraction of the total cohort.

During their hospital stay, 225 neonates (88.2%) were tested for SARS-CoV-2 and 5 (2.2%) had positive results (Table 2). Thus, the test result positivity rate was 2.2% (95% CI, 1.0-5.1) among newborns tested before hospital discharge. A total of 124 newborns (55.1% of those tested) underwent only 1 test during their birth hospitalization. Including all 6 neonates with positive SARS-CoV-2 test results within the first week of life (eTable 1 in the Supplement), the test result positivity rate was 2.7% among those who were tested. Two neonates who presented with respiratory distress were delivered preterm: 1 newborn had nasal congestion, and 3 newborns were asymptomatic. We found no substantial differences in maternal or neonatal characteristics between neonates who were tested and those who were not during their hospitalization (eTable 2 in the Supplement).

### Risk Factors Associated With Neonatal Test Result Positivity

Characteristics of neonates with negative and those with positive SARS-CoV-2 test results are shown in eTable 3 in the Supplement. In unadjusted analyses, neonates with positive test results were more likely to be born to mothers with symptomatic COVID-19 (OR, 1.84; 95% CI, 0.51-6.58; *P* = .35), less likely to be delivered vaginally (OR, 0.39; 95% CI, 0.12-1.22; *P* = .11), and less likely to room-in (OR, 0.26; 95% CI, 0.02-3.07; *P* = .29), but none of these results was statistically significant (Table 3). Individual-level racial/ethnic minority status or non–English-speaking status was not associated with a higher risk of the newborn having a positive test result (OR, 0.81; 95% CI, 0.09-7.66; *P* = .85). Adjusting for maternal symptoms, delivery mode, and rooming-in practice, mothers with high SVI (≥90th percentile) were more likely to have neonates with positive SARS-CoV-2 test results (adjusted OR, 4.95; 95% CI, 1.53-16.01; *P* = .008) (Table 3).

**Table 3.** Factors Associated With Positive SARS-CoV-2 Test Results Among 226 Neonates^a^

Variable	Unadjusted		Adjusted	
	OR (95% CI)	*P* value	OR (95% CI)	*P* value
Maternal SVI ≥90th percentile vs other	5.49 (2.11-14.29)	.001	4.95 (1.53-16.01)	.008
Maternal symptomatic vs asymptomatic COVID-19	1.84 (0.51-6.58)	.35	0.71 (0.49-1.02)	.07
Vaginal vs cesarean delivery	0.39 (0.12-1.22)	.11	0.47 (0.16-1.40)	.18
Any vs no rooming-in	0.26 (0.02-3.07)	.29	0.29 (0.04-2.29)	.24
Racial/ethnic minority or non–English speaker vs other	0.81 (0.09-7.66)	.85	NA	NA

Abbreviations: NA, not applicable; OR, odds ratio; SVI, Social Vulnerability Index.

^a^ Mixed-effects regression models accounted for hospital clustering using a robust sandwich estimator. Positive test occurred in the first 5 days after delivery. This analysis includes 6 newborns with a positive SARS-CoV-2 test result within the first week of life (5 positive results during the birth hospitalization, and 1 positive result after discharge on day 5 of life).

### Adverse Neonatal Health Outcomes and Maternal Delivery Indication

Table 4 shows that all adverse neonatal health outcomes (preterm or low birth weight, very preterm or very low birth weight, delivery room resuscitation, CPAP or mechanical ventilation, and length of stay) were increased among neonates who were born to mothers whose worsening COVID-19 illness led to a delivery indication. Of 62 preterm births, 17 (27.4%) were indicated by maternal worsening symptoms, but we did not otherwise collect data on the indication for preterm delivery. Among the neonates who were delivered for worsening maternal illness, 17 (73.9%) were delivered preterm.

**Table 4.** Adverse Neonatal Health Outcomes by Delivery Indication

Newborn health indicator	No. (%)		*P* value^a^
	Delivery indication		
	Worsening maternal COVID-19 illness (n = 23)	Other (n = 232)	
Delivery room resuscitation^b^	14 (60.9)	35 (15.1)	<.001
Preterm birth (<37 wk) or LBW (<2500 g)	17 (73.9)	45 (19.4)	<.001
Very preterm birth (<32 wk) or VLBW (<1500 g)	7 (30.4)	10 (4.3)	<.001
Respiratory support^c^	15 (65.2)	25 (10.8)	<.001
Therapeutic hypothermia	1 (4.4)	3 (1.3)	.17
Newborn LOS, d			
Mean (SD)	17.6 (24.0)	5.1 (8.5)	<.001
Median (IQR)	9.5 (4-18)	3 (2-4)	<.001

Abbreviations: IQR, interquartile range; LBW, low birth weight; LOS, length of stay; VLBW, very low birth weight.

^a^ *P* values account for hospital-level clustering.

^b^ Delivery room resuscitation includes positive-pressure ventilation, intubation, and/or chest compressions.

^c^ Respiratory support is continuous positive airway pressure or mechanical ventilation.

### Newborn Follow-up Data 30 Days After Hospital Discharge

Of the 255 neonates who were exposed to SARS-CoV-2, 151 (59.2%) had at least 1 postdischarge medical encounter documented in the EMR (Table 5). The demographic characteristics were similar for neonates with and those without postdischarge EMR information except the month of birth differed significantly between the 2 groups (eTable 4 in the Supplement). Most encounters were for routine care, whereas 28 were nonroutine visits, including 18 visits to urgent care or an emergency department. None of the neonates who received routine care only underwent testing for SARS-CoV-2, whereas 7 neonates with nonroutine encounters were tested; 1 had a positive SARS-CoV-2 test result on day 5 of life during an emergency department visit for nasal congestion (eTable 1 in the Supplement). Four neonates were rehospitalized in the first 30 days after discharge, and none was for conditions directly associated with SARS-CoV-2 infection (eTable 5 in the Supplement).

**Table 5.** Follow-up Data of Neonates in the First 30 Days After Hospital Discharge

30-d Postdischarge data	No. (%)
Total No. of neonates with ≥1 encounter	151 (59.2)
Neonates with SARS-CoV-2 testing	7 (4.6)
Positive result among neonates with known follow-up encounters	1 (0.7)
Positive result among neonates tested	1 (14.3)
Neonates with asymptomatic visits only	123 (81.5)
SARS-CoV-2 test sent	0
Neonates with any symptomatic visits	28 (18.5)
SARS-CoV-2 test sent	7 (25.0)
Location of visit	
Pediatric clinic	23 (82.1)
ED or urgent care	18 (64.3)
Rehospitalization	4 (14.3)
Telehealth	3 (10.7)
Other	2 (7.1)
Complaint type	
Gastrointestinal (emesis, constipation)	4 (14.3)
Respiratory	8 (28.6)
Fever	1 (3.6)
Lethargy, poor feeding	1 (3.6)
Other or newborn-related^a^	9 (32.1)

Abbreviation: ED, emergency department.

^a^ Fall, fussiness, rash, and spit-up.

## Discussion

To our knowledge, this study presented data for the largest US cohort of neonates who were born to mothers with positive SARS-CoV-2 test results. Among the 255 mother-newborn dyads from 11 hospitals in Massachusetts, we found a 2.2% test result positivity rate in neonates who underwent SARS-CoV-2 testing during birth hospitalization. In addition, we identified maternal social vulnerability, as defined by zip code, as a risk factor for neonatal test result positivity, whereas maternal COVID-19 symptoms, delivery mode, and rooming-in practice were not significant factors. Adverse health outcomes among neonates who were born to mothers with positive SARS-CoV-2 test results were associated with delivery prompted by worsening maternal COVID-19 illness, whereas the health outcomes of neonates with positive SARS-CoV-2 test results were largely favorable. We identified minimal neonatal health burden that could be directly associated with SARS-CoV-2 infection within 30 days after hospital discharge.

Previous studies reported a wide range in the percentage of test result positivity among neonates who were born to mothers with positive SARS-CoV-2 test results. Systematic reviews reported a 3.1% to 9.1% neonatal test result positivity, but these reviews were prone to publication bias and included studies that were published before the wide implementation of maternal surveillance testing, potentially overestimating the true incidence of positive SARS-CoV-2 test results in neonates.^5,6^ In contrast, 3 New York City cohorts with larger sample sizes reported virtually no neonatal test result positivity despite high rates of rooming-in and direct breastfeeding.^8,9,18^ Given that 30 newborns in the present cohort were not tested for SARS-CoV-2, it is possible that the neonatal test result positivity rate is even lower than 2.2%. Overall, the literature suggests low rates of acquired infection among New York City and Massachusetts neonates who were born to mothers with positive SARS-CoV-2 test results during the first wave of the pandemic.^9,18^

The COVID-19 pandemic has disproportionately affected Hispanic and Black communities with higher infection, morbidity, and mortality rates.^26,27^ In children, higher SVI, Hispanic ethnicity, and Black race independently increased the risk of multisystem inflammatory syndrome.^28^ We found that high social vulnerability, defined by the maternal zip code, was associated with a nearly 5-fold higher risk for neonatal test result positivity, although individual-level race/ethnicity and language status were not associated with a higher risk for neonatal test result positivity. Previous studies have identified the built environment and other neighborhood variables as factors associated with SARS-CoV-2 infection in pregnant women^29^ and adverse perinatal outcomes in general,^30,31^ but we could find no published studies that examined the sociodemographic risk factors for test result positivity among neonates who were exposed to SARS-CoV-2. The association of maternal social vulnerability with neonatal test result positivity was only slightly attenuated by adjustments for maternal illness severity, suggesting that nonclinical factors may be at play. We speculate that living in a socially disadvantaged neighborhood may be a factor in stress-mediated alterations in the maternal and/or fetal immune response, facilitating SARS-CoV-2 transmission.^14,15,32^ Given that 4 of 6 neonates with positive results were born at the same hospital, it is possible that hospital-level factors, such as air flow or building design, were also at play, although we minimized the impact of hospital-level factors by accounting for clustering in the multivariate model.

Newborns with positive SARS-CoV-2 test results appeared to have minimal burden of illness that was directly associated with a viral infection. However, those who were born in the context of delivery prompted by worsening maternal COVID-19 symptoms were more likely to be preterm births, which led to a need for resuscitation in the delivery room, CPAP or mechanical ventilation, and longer length of stay. These results indicate that maternal SARS-CoV-2 infection has an association with neonatal health, which is brought about not through viral transmission from the mother to the neonate but rather through the impact of preterm delivery undertaken because of the mother’s worsening illness.

Few previous studies have ascertained the neonatal outcomes beyond the birth hospitalization. We leveraged EMR data to identify nonroutine newborn health care visits possibly related to SARS-CoV-2 infection. Reassuringly, we found very few such encounters. The findings in this study complement those in the US-based PRIORITY (Pregnancy Coronavirus Outcomes Registry) study, which involved maternal reporting of newborn outcomes through 6 to 8 weeks of age.^17^ In the PRIORITY study, 2 of 80 neonates presented with upper respiratory tract infection symptoms, 0 had pneumonia, and 0 was rehospitalized; 2 had positive SARS-CoV-2 test results during the follow-up period.^17^ In addition, a New York City study with routine clinical follow-up for all neonates exposed to SARS-CoV-2 reported no substantial SARS-CoV-2–related illness up to 1 month after hospital discharge.^18^ Overall, we did not observe a substantial burden of SARS-CoV-2 among neonates up to 1 month after hospital discharge, although viral testing was limited in this study.

### Strengths and Limitations

This study has some strengths. We included 11 academic and community hospitals, representing more than 50% of all births in Massachusetts, but the findings may not generalize to nonacademic and level I or II hospitals. Racial and ethnic diversity of the study population was commensurate with the population in a recent report by the Centers for Disease Control and Prevention of pregnant women in the US with SARS-CoV-2,^33^ suggesting generalizability to other US perinatal populations. We leveraged active hospital-level surveillance for COVID-19 but may have missed a small number of dyads.

This study has some limitations. Like other studies,^34^ the present study had limited ability to differentiate transient colonization from true positive test results in newborns because of a lack of repeated neonatal testing. In addition, some neonates were not tested during the birth hospitalization, and few were tested after discharge. Because of our reliance on clinical SARS-CoV-2 testing data, we could not determine the exact timing of maternal infection, especially in mothers with asymptomatic COVID-19. Although the study sample was large compared with samples in other published studies, we had limited ability to examine multiple factors simultaneously because only 6 newborns had positive results; residual confounding was possible. Practices evolved during the study period such that, by month 3, rooming-in and breastfeeding were standard in most, if not all, hospitals in Massachusetts. Furthermore, the evolution of these practices did not vary by social factors.^23^ We were not able to ascertain 30-day outcome data for all neonates because of the limitations of EMR-based follow-up. Demographic characteristics were similar in mothers and neonates who had available EMR encounters vs those who did not, but the data on 30-day outcomes may not be missing at random, preventing firm conclusions.

## Conclusions

The neonatal test result positivity rate for SARS-CoV-2 during the birth hospitalization was 2.2% in a statewide perinatal cohort. Maternal social vulnerability was associated with an increased risk for neonatal test result positivity, whereas individual-level maternal race/ethnicity and language status was not. Newborns who had exposure to SARS-CoV-2 were at risk for both direct and indirect adverse health outcomes, whereas preterm delivery owing to worsening maternal COVID-19 illness was associated with substantial neonatal morbidity. The findings support ongoing surveillance of the virus and long-term follow-up.

## Notes

Supplement. **eFigure.** Participating MA Hospitals, Level of Care, and Yearly Births **eTable 1.** MA Newborn Cases With Positive SARS-CoV-2 Testing Born to Mothers With SARS-CoV-2 **eTable 2.** Characteristics of Newborns Who Were Tested vs Not During Their Hospital Stay **eTable 3.** Characteristics of Newborns With Negative and Positive SARS-CoV-2 Test(s) **eTable 4.** Characteristics of Newborns With and Without Post-Discharge EMR Information **eTable 5.** Newborn Cases Who Were Re-Hospitalized Within 30 Days From Birth Hospital Discharge
