# Supplementary material for: Association of Maternal Perinatal SARS-CoV-2 Infection With Neonatal Outcomes During the COVID-19 Pandemic in Massachusetts
Source: JAMA Netw Open. 2021 Apr 23;4(4):e217523. doi: 10.1001/jamanetworkopen.2021.7523 (PMC8065376; doi:10.1001/jamanetworkopen.2021.7523)
Supplement: Supplement. — eFigure. Participating MA Hospitals, Level of Care, and Yearly Births eTable 1. MA Newborn Cases With Positive SARS-CoV-2 Testing Born to Mothers With SARS-CoV-2 eTable 2. Characteristics of Newborns Who Were Tested vs Not During Their Hospital Stay eTable 3. Characteristics of Newborns With Negative and Positive SARS-CoV-2 Test(s) eTable 4. Characteristics of Newborns With and Without Post-Discharge EMR Information eTable 5. Newborn Cases Who Were Re-Hospitalized Within 30 Days From Birth Hospital Discharge [file jamanetwopen-e217523-s001.pdf]

Supplementary Online Content

Angelidou A, Sullivan K, Melvin PR, et al. Association of maternal perinatal SARS-CoV-2 infection with neonatal outcomes during the COVID-19 pandemic in Massachusetts. *JAMA Netw Open*. 2021;4(4):e217523. doi:10.1001/jamanetworkopen.2021.7523

eFigure. Participating MA Hospitals, Level of Care, and Yearly Births

eTable 1. MA Newborn Cases With Positive SARS-CoV-2 Testing Born to Mothers With SARS-CoV-2

eTable 2. Characteristics of Newborns Who Were Tested vs Not During Their Hospital Stay

eTable 3. Characteristics of Newborns With Negative and Positive SARS-CoV-2 Test(s)

eTable 4. Characteristics of Newborns With and Without Post-Discharge EMR Information

eTable 5. Newborn Cases Who Were Re-Hospitalized Within 30 Days From Birth Hospital Discharge

This supplementary material has been provided by the authors to give readers additional information about their work.

© 2021 Angelidou A et al. *JAMA Network Open.*

eFigure. Participating MA Hospitals, Level of Care and Yearly Births

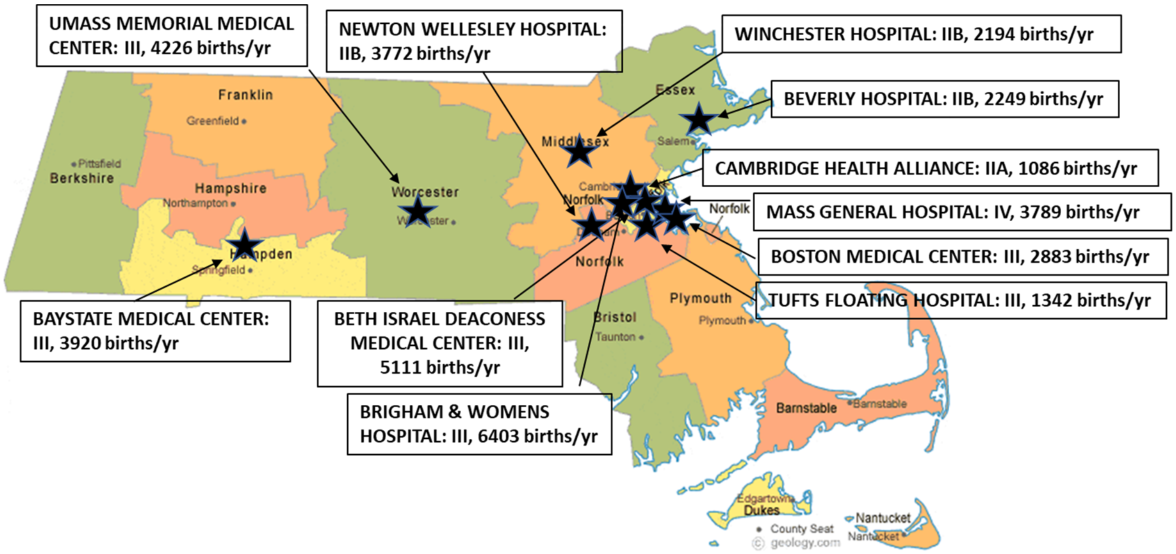

Source: Massachusetts Department of Public Health, 2017.

© 2021 Angelidou A et al. *JAMA Network Open.*

eTable 1. **MA Newborn Cases with Positive SARS-CoV-2 Testing Born to Mothers With SARS-CoV-2.**

Testing period	In-hospital					After discharge
Case number	Case 1	Case 2	Case 3	Case 4	Case 5	Case 6
Hospital	A	A	A	B	C	A
Maternal race/ethnicity	Hispanic	Non-Hispanic Black	Hispanic	Non-Hispanic White	Non-Hispanic Other	Hispanic
Maternal language	English	English	Spanish	English	Spanish	Spanish
SVI ≥90th percentile	Yes	No	Yes	Yes	No	Yes
Maternal COVID-19 symptoms at delivery	Yes	Yes	Yes	No	No	Yes
Mode of delivery	Cesarean	Cesarean	Cesarean	Cesarean	Vaginal	Vaginal
Preterm infant	Yes	Yes	No	No	No	No
Gestational Age	35 wk 1 d	35 wk 1 d	38 wk 0 d	38 wk 0 d	41 wk 0 d	40 wk 2 d
Timing of infant testing	HD1/HD2/HD5	HD1/HD2/HD4/HD5	HD1/HD2/HD5	HD3	HD2	HD1/HD2/ED D5
Sequence of infant testing	+/+/+	-/inconclusive/invalid/+	-/+/-	+	+	-/-/+
Neonatal signs	Respiratory distress, feeding immaturity	Respiratory distress	None	None	None	Nasal congestion, rhinorrhea, sneezing
Any rooming-in	No	No	Yes	Yes	Yes	No
Any direct breastfeeding	No	No	Yes	No	Yes	No

Abbreviations: ED, Emergency Department; HD, Hospital Day; NHB, non-Hispanic Black; NHW, non-Hispanic White; SVI, Social Vulnerability Index

eTable 2. Characteristics of Newborns Who Were Tested vs Not During Their Hospital Stay.

Variable	Infants tested during hospital stay (N= 225)	Infants not tested during hospital stay (N=30)	*P* Value[a]
**Social factors**			
Maternal race/ethnicity, No. (%)			.20
Hispanic, any race	110 (48.9)	13 (43.3)	
Non-Hispanic white	40 (17.8)	10 (33.3)	
Non-Hispanic black	43 (19.1)	5 (16.7)	
Non-Hispanic other	32 (14.2)	2 (6.7)	
Black race or Hispanic ethnicity, No. (%)			.38
Yes	153 (68.0)	18 (60.0)	
No	72 (32.0)	12 (40.0)	
Maternal language status, No. (%)			.24
English	117 (52.0)	19 (63.3)	
Non-English	108 (48.0)	11 (36.7)	
Maternal SVI, No. (%)			.66
SVI ≥90th percentile	61 (27.1)	7 (23.3)	
<90th percentile	164 (72.9)	23 (76.7)	
**Pregnancy and delivery factors**			
Maternal illness, No. (%)			.54
Symptomatic	70 (31.1)	11 (36.7)	
Asymptomatic	155 (68.9)	19 (63.3)	
Cesarean delivery, No. (%)			.51
Yes	98 (43.6)	15 (50.0)	
No	127 (56.4)	15 (50.0)	
Preterm birth (<37 wk), No. (%)			.12
Yes	50 (22.2)	3 (10.0)	
No	175 (77.8)	27 (90.0)	
**Newborn Characteristics**			
Month of Birth, No. (%)			.95
March	15 (6.7)	2 (6.7)	
April	68 (30.2)	11 (36.7)	
May	78 (34.7)	10 (33.3)	
June	50 (22.2)	5 (16.7)	
July	14 (6.2)	2 (6.7)	
Preterm (< 37 wk) or LBW (< 2500 g), No. (%)			.05
Yes	59 (26.2)	3 (10.0)	
No	166 (73.8)	27 (90.0)	

© 2021 Angelidou A et al. *JAMA Network Open.*

Resuscitation at Birth, No. (%)			.56
Drying and stimulation	177 (78.7)	25 (83.3)	
Oxygen, positive pressure of any kind, intubation, or chest compressions	48 (21.3)	5 (16.7)	

Abbreviations: LBW, low birth weight; SVI, Social Vulnerability Index.
[a]*P* values account for hospital clustering using Cochran-Mantel-Haenszel chi-square tests.

© 2021 Angelidou A et al. *JAMA Network Open.*

eTable 3. Characteristics of Newborns with Negative and Positive SARS-CoV-2 Test(s).

Variable	Infants with negative test (N= 220)	Infants with positive test (N=6)	*P* Value[a]
**Social factors**			
Maternal race/ethnicity, No. (%)			.97
Hispanic, any race	108 (49.1)	3 (50.0)	
Non-Hispanic white	39 (17.7)	1 (16.7)	
Non-Hispanic black	42 (19.1)	1 (16.7)	
Non-Hispanic other	31 (14.1)	1 (16.7)	
Black race or Hispanic ethnicity, No. (%)			.48
Yes	150 (68.2)	4 (66.7)	
No	70 (31.8)	2 (33.3)	
Maternal language status, No. (%)			.71
English	114 (51.8)	3 (50.0)	
Non-English	106 (48.2)	3 (50.0)	
Maternal SVI, No. (%)			.05
SVI ≥90th percentile	57 (25.9)	4 (66.7)	
<90th percentile	163 (74.1)	2 (33.3)	
**Pregnancy and delivery factors**			
Maternal illness, No. (%)			.50
Symptomatic	68 (30.9)	3 (50.0)	
Asymptomatic	152 (69.1)	3 (50.0)	
Cesarean delivery, No. (%)			.28
Yes	94 (42.7)	4 (66.7)	
No	126 (57.3)	2 (33.3)	
Preterm birth (<37 wk), No. (%)			.26
Yes	49 (22.3)	2 (33.3)	
No	171 (77.7)	4 (66.7)	
**Hospital practices**			
Any rooming-in, No. (%)			.07
Yes	142 (64.5)	2 (33.3)	
No	78 (35.5)	4 (66.7)	
Any direct breastfeeding, No. (%)			.08
Yes	127 (57.7)	2 (33.3)	
No	93 (42.3)	4 (66.7)	

Abbreviations: SVI, Social Vulnerability Index.
[a]*P* values account for hospital clustering using Cochran-Mantel-Haenszel chi-square tests.

© 2021 Angelidou A et al. *JAMA Network Open.*

eTable 4. Characteristics of Newborns With and Without Post-Discharge EMR Information.

	Any Infant Post-Discharge Encounter?		
**Variable**	**No (N=104)**	**Yes (N=151)**	***P* value[a]**
**Maternal Demographics/Social Factors**			
Race/Ethnicity, No. (%)			
Underrepresented Minority (Hispanic or Black)	60 (57.7)	111 (73.5)	.84
Language, No. (%)			
English	66 (63.5)	70 (46.4)	.52
Spanish	27 (26.0)	57 (37.7)	
Other	11 (10.6)	24 (15.9)	
Maternal Age, mean (SD), y	30.1 (6.0)	30.5 (6.5)	.58
**Newborn Characteristics**			
Inborn, No. (%)	101 (97.1)	146 (96.7)	.69
Female, No. (%)	56 (53.8)	75 (49.7)	.87
Month of Birth, No. (%)			
March	11 (10.6)	6 (4.0)	**.002**
April	26 (25.0)	53 (35.1)	
May	31 (29.8)	57 (37.7)	
June	28 (26.9)	27 (17.9)	
July	8 (7.7)	8 (5.3)	
Preterm (< 37 wk) or LBW (< 2500 g), No. (%)	26 (25.0)	36 (23.8)	.07
Resuscitation at Birth, No. (%)			
Drying and stimulation	79 (76.0)	123 (81.5)	.75
Oxygen, positive pressure of any kind, intubation, or chest compressions	25 (24.0)	28 (18.5)	

Abbreviations: LBW, low birth weight.
[a]*P* values account for hospital clustering using Cochran-Mantel-Haenszel chi-square tests.

© 2021 Angelidou A et al. *JAMA Network Open.*

eTable 5. Newborn Cases Who Were Re-Hospitalized Within 30 Days From Birth Hospital Discharge.

	Case 1	Case 2	Case 3	Case 4
**Clinical synopsis**	Presented to the ED on day of life 3 with hematemesis, had an apneic/bradycardic episode and was admitted for full sepsis evaluation. Was discharged after 48 hours of antibiotics, blood culture subsequently returned positive for micrococcus luteus thought to be a contaminant	Admitted at 2 weeks of life for initiation of propranolol for a ventricular arrhythmia	Admitted for hyperbilirubinemia due to G6PD deficiency	Admitted for respiratory distress and was subsequently diagnosed with laryngomalacia
**Viral testing done**	No	Yes	No	Yes
**Viral test results**	NA	Negative	NA	Negative

Abbreviations: NA, Not applicable.

© 2021 Angelidou A et al. *JAMA Network Open.*
